# When tests don’t fit the rules: regulatory challenges for agnostic metagenomic next-generation sequencing in infectious diseases diagnostics

**DOI:** 10.1128/jcm.00987-26

**Published:** 2026-09-10

**Authors:** Nick P. G. Gauthier, Vishal Kapoor, David C. Gaston, Neha Prasad, Precious Nwaobia, Colleen S. Kraft, Dev Mittar

**Affiliations:** 1 BugSeq Bioinformatics Inc., Vancouver, Canada; 2 Columbia University 5798, New York, New York, USA; 3 Vanderbilt Health 482981, Nashville, Tennessee, USA; 4 ASM, Washington, DC, USA; 5 Emory University 1371, Atlanta, Georgia, USA; Medical College of Wisconsin, Milwaukee, Wisconsin, USA

**Keywords:** clinical interpretation, infectious disease diagnostics, regulatory science, agnostic diagnostic, mNGS, clinical metagenomics

## Abstract

Metagenomic next-generation sequencing (mNGS) enables agnostic detection of bacteria, viruses, fungi, and parasites from clinical specimens. Patient access is restricted due to their current regulatory classification as Laboratory Developed Tests, which are offered by a limited number of laboratories. While patient access may be expanded through Food and Drug Administration authorization of mNGS-based *in vitro* diagnostics, some regulatory requirements for achieving this classification can be challenging to apply due to the agnostic nature of mNGS. For example, existing guidelines recommend organism-by-organism performance characterization similar to targeted molecular tests involving fixed analytes with stable detection thresholds. This minireview synthesizes validation and regulatory challenges unique to mNGS-based diagnostics and describes how laboratories and developers currently navigate available pathways. We focus on core analytical and clinical validation challenges, including discussion on the central role of bioinformatics pipelines and reference databases. We then summarize practical considerations for regulatory engagement, labeling, clinical-facing reporting, and alignment of mNGS assays to existing quality system frameworks. Finally, we outline future oversight considerations, including the need to distinguish mNGS from multiplexed PCR paradigms and the potential value of modular, component-based regulatory approaches that separately evaluate reagents, instruments, software, and databases, enabling system-level oversight rather than organism-by-organism validation. Together, these considerations aim to support risk-appropriate, scalable oversight, and accelerate adoption of mNGS in clinical microbiology.

## WHY mNGS DOESN’T FIT EXISTING REGULATORY MODELS

Unlike conventional molecular diagnostics that target predefined pathogens associated with a specific clinical syndrome, metagenomic next-generation sequencing (mNGS) enables broad, agnostic detection of microbial nucleic acids from bacteria, viruses, fungi, and parasites. As a result, mNGS can identify pathogens that may not have been initially suspected based on the presenting syndrome or clinical differential diagnosis or are unable to be cultivated. Some clinical laboratories have successfully implemented mNGS-based laboratory-developed tests (LDTs) for diagnosing infections in cerebrospinal fluid (CSF), plasma cell-free DNA, and respiratory samples (1–3). However, mNGS tests have not yet received market authorization as an FDA-regulated *in vitro* diagnostic (IVD), thereby limiting scalability, reimbursement, and sustainable patient access (4–12).

This divergence reflects fundamental structural differences between the regulations for LDTs under the Clinical Laboratory Improvement Amendments of 1988 (CLIA) and those for IVDs under the Food and Drug Administration (FDA). Under CLIA, laboratories—not products—are regulated. Validation of the LDT is contextual to the specific laboratory, with laboratory directors, clinicians, and institutional governance structures responsible for the appropriate interpretation of results. This model is structurally tolerant of agnostic diagnostics, as risk is mitigated locally. In contrast, the FDA regulates devices intended for use at scale under decontextualized conditions, requiring uncertainty to be bound in advance to mitigate population-level risks, such as misdiagnosis, overdiagnosis, or underdiagnosis. Therefore, expanding patient access to mNGS-based diagnostics will require alignment with FDA’s regulatory frameworks, which are typically defined through iterative engagement with developers.

Current regulatory frameworks and validation conventions for infectious disease diagnostics are tailored to targeted molecular assays, like qPCR, with predefined analytes and fixed analytical characteristics. Those assumptions do not adequately translate to mNGS-based diagnostics, where performance depends on background nucleic acids, sequencing technologies, sequencing depth, bioinformatics algorithms, evolving reference databases, and additional analytic factors. Effectiveness in informing clinical decision-making also relies on proper interpretation of results, which is relatively straightforward for targeted assays with predefined analytes and thresholds, but more complex for mNGS assays where clinically relevant signal must be distinguished from background noise. As a result, validation approaches that work well for targeted assays with predefined analytes do not easily translate to pathogen-agnostic mNGS diagnostics. Several groups have proposed frameworks and considerations addressing validation, reporting, interpretation, quality systems, and translational implementation of metagenomic sequencing across a range of application domains, including the STRATUM framework (13). However, important questions remain regarding the regulatory evaluation and implementation of patient-facing pathogen-agnostic infectious disease diagnostics.

This minireview focuses on practical challenges and emerging approaches to support the development and use of mNGS diagnostics as IVDs in the U.S. healthcare system. Rather than proposing new formal guidance, we describe current regulatory navigation strategies and outline future considerations that could reduce uncertainty for laboratories, developers, regulators, and public health stakeholders.

## VALIDATION CHALLENGES UNIQUE TO MNGS

### Infeasibility of comprehensive clinical validation

Many analytical validation expectations commonly applied to targeted molecular assays become difficult to translate to mNGS systems. The analytical performance characteristics for targeted molecular tests are usually established for a representative set of pathogens, which is not appropriate for mNGS-based tests. To similarly validate the performance of an mNGS-based assay, a much larger set of pathogens would be required to capture the agnostic capability of mNGS to detect a disease-causing pathogen that aligns with the clinical presentation. Even if a closely related species to a validated representative target is detected, such a finding cannot be officially reported under current regulatory guidelines for IVD molecular infectious disease assays. However, analytical validation of an exhaustive list of pathogens is impractical and cost-prohibitive for developers.

Existing validation guidelines for targeted molecular diagnostics also do not account for the utility of mNGS-based tests in detecting rare or emerging infectious disease threats. Traditional approaches rely on comparison to a gold standard and require a significant number of positive samples per analyte. However, this model becomes impractical for mNGS systems that can detect rare pathogens, many of which lack established comparator methods. Rare pathogens, including amoebic encephalitis pathogens, emerging zoonotic viruses, and geographically restricted fungal pathogens, may never be observed in sufficient numbers to support statistical validation, and for novel organisms, there is no ground truth at all. As a result, standard performance metrics such as sensitivity, specificity, and positive/negative percent agreement cannot always be meaningfully calculated at the individual-organism level (2, 14–17).

Recognizing that traditional validation paradigms may require adaptation for broad-range infectious disease diagnostics, the FDA issued draft guidance in 2016 (18), which was later withdrawn. The agency has since emphasized more flexible, case-specific feedback mechanisms through Q-submissions and indicated that validation strategies can be tailored to intended use, including representative organism approaches rather than exhaustive organism-by-organism validation.

In situations where no single comparator method can definitively establish the presence or absence of infection, alternative approaches have been pursued for evaluating clinical performance. Composite reference standards, which may integrate microbiology results, orthogonal molecular testing, histopathology, imaging findings, and clinical adjudication, have been used when a single gold standard is unavailable. Similar approaches may also be useful for validating mNGS diagnostics—particularly for rare pathogens, novel organisms, or clinical scenarios where conventional diagnostic methods have poor analytical performance characteristics.

One potential approach would be to select representative organisms based on biological and technical characteristics that influence assay performance when organism-specific performance evaluation is impractical. For example, viral validation panels could include both RNA and DNA viruses, while bacterial validation panels could include Gram-positive and Gram-negative bacteria, as well as mycobacteria. Similar considerations may apply to fungal and parasitic pathogens. Additional factors such as genome size, nucleic acid extraction efficiency, target abundance, and specimen type may also influence the selection of representative organisms and support broader performance claims.

### Analyte detection thresholds

In the context of viral diagnostics, product developers have sometimes been directed to use frameworks developed for respiratory viral panels as reference points. While such special controls guidance provides a useful benchmark for targeted assays, its underlying assumptions do not fully translate to mNGS tests. For instance, targeted qPCR performance is often characterized per analyte, with assay-intrinsic specificity derived from primer/probe design and analyte-specific fixed LoD estimates (19–22). Rather than a cycle number at which fluorescence crosses a defined threshold, mNGS produces continuous measures such as read counts (e.g., reads per million [RPM]) or relative abundance (e.g., 0.005% of microbial reads assigned to a particular organism). Because mNGS simultaneously interrogates all nucleic acids present within a specimen rather than a predefined set of targets, low-level contaminant reads originating from reagents, laboratory environments, sample handling, or sequencing workflows may be more difficult to distinguish from clinically relevant organisms. Therefore, a positive detection call for mNGS tests must be inferred from multiple variables, including read abundance, genomic coverage, extraction efficiency, background nucleic acid content, organism viability, software algorithms, and reference databases.

While environmental and reagent contamination are recognized challenges across molecular diagnostic technologies, their impact may be amplified in agnostic sequencing workflows. Setting a threshold to distinguish true positives from background noise requires accounting for multiple factors, including sequencing depth, coverage, run-specific negative controls, and microbial contaminants found in reagents, also known as reagent microbiome or kitome, which consists of microbial nucleic acids introduced through extraction kits, library preparation reagents, and other laboratory consumables (23, 24). Phenomena such as read bleed-over, cross-contamination, and index hopping further complicate interpretation. While contamination in targeted PCR assays is typically evaluated against predefined analytes and fixed reporting thresholds, mNGS interpretation often requires distinguishing true pathogens from low-level contaminant signals across thousands of potential taxa simultaneously (25–32).

All these factors, independently or collectively, create a nonzero baseline of microbial reads even in sterile samples. In some cases, low-level contamination exceeds the set positivity thresholds, leading to false positives that are indistinguishable from the true signal. Existing FDA carryover guidance, which focuses on between-run contamination, does not address these within-run effects, and there are no established standards for acceptable index hopping rates or methods for characterizing and mitigating kitome contamination. Thus, there is no consensus on whether thresholds should be absolute or relative, nor is there FDA guidance on how to validate such adaptive decision rules.

### Data normalization with controls

While the inclusion of internal and external process controls is critical for elucidating true pathogen signals from background noise in bioinformatic outputs, choosing which control materials to include requires careful consideration. Key considerations include (i) the nucleic acid molecule sequenced (i.e., DNA, RNA, or both), (ii) which parts of the sample processing workflow the control is designed to control for, and (iii) the underlying background nucleic acid concentration for the intended specimen type that may influence control concentration. Leveraging these controls for data normalization and quality control is critical. Several mNGS guidelines and validated LDTs set examples of normalizing to or detecting internal or negative controls as reporting criteria (2, 33–35). While specific control normalization thresholds are useful for individual mNGS-based LDTs, the variability of sample processing techniques, reagents, sequencing technologies, and depths, and bioinformatic methods preclude widespread standardization of reporting criteria and translation of mNGS to smaller institutions that do not have the capacity to generate the evidence needed to establish robust mNGS reporting criteria.

Though biologic standards may enable sample processing device, sequencing, or bioinformatic manufacturers to evaluate upstream or downstream components of the mNGS workflow outside their control, it is unclear whether regulatory bodies will accept this rationale, which would enable manufacturers to make broad claims about the intended use of their devices. Detailed regulatory guidance on NGS assay controls for IVD manufacturers will expedite the translation of mNGS-based diagnostics to settings that lack the resources to validate an LDT and require pre-validated solutions. Future guidelines should account for the nuances of control selection and utilization in NGS-based IVDs compared with other molecular diagnostic technologies, such as PCR.

For developers pursuing clinical implementation of mNGS, validation strategies may benefit from focusing on representative organism groups rather than exhaustive organism-by-organism testing. While the FDA accepts that comprehensive validation of all pathogens detectable by mNGS tests is infeasible, early engagement with regulatory agencies may help align validation expectations with intended-use claims. Risk mitigation approaches should include standardized contamination monitoring, assessment of index hopping and carryover effects, transparent reporting thresholds, implementation of controls, and predefined procedures for evaluating changes to analytical workflows. These approaches may help improve consistency while preserving the flexibility needed for agnostic diagnostics (Table 1).

**TABLE 1:** Potential laboratory quality control and documentation elements for mNGS assays^*a*^

Category	Sample element
Test & specimen context	Intended use/clinical syndrome
	Specimen type and source (e.g., CSF, plasma, bronchoalveolar lavage)
	Assay reporting scope (pathogen ID only vs ID +AMR markers)
	Device, software, and database unique identifiers and versions
	Definition of algorithms for the classification of potential pathogenic and commensal organisms
	Analytical performance disclaimers
Workflow metadata (high level)	Nucleic acid type sequenced (DNA/RNA/both)
	Enrichment and/or depletion strategy (shotgun, capture, amplicon, etc.)
	Sequencing platform and device
	Repeat testing status, if applicable
Quality control summary	Sample adequacy metrics (host background reads, total library yield, total number of reads, etc.)
	Run-level controls included and status (e.g., negative, positive, internal)
	Statement of qualitative QC pass/fail and limitations
Bioinformatics & reference transparency	Pipeline name/version
	Reference database name/version
	Date of last database update and change log

^*a*^ Elements shown are examples and should not be interpreted as universally required quality control or documentation metrics.

## BIOINFORMATICS, REFERENCE DATA, AND CHANGE MANAGEMENT

### Can *in silico* studies fill the gap?

To resolve the analytical and clinical validation challenges for mNGS-based tests, independent bioinformatic validation can be pursued alongside the required *in vitro* validation to ensure the accuracy of the analytic workflow and reporting thresholds. In the past, regulatory bodies have accepted *in silico* validation for molecular diagnostic assays to support validation of certain test performance metrics (e.g., inclusivity, cross-reactivity). For validating NGS-based diagnostics, *in silico* validation leveraging simulated contrived data sets with established standards may help support the broader claims of agnostic diagnostics. Such analyses could be performed by assay developers or bioinformatics pipeline manufacturers as part of formal analytical validation of the device, using well-characterized reference data sets derived from simulated or experimental sequence data to evaluate performance across a broad range of organisms. This approach could aid in the generation of performance data for pathogens beyond the initial set of representative organisms for which diagnostic performance is supported by *in vitro* analytical validation data (36). Ultimately, end-to-end workflow validation of the combined bench and bioinformatic approaches is necessary.

### Reference database reliability

Two key challenges exist with regard to *in silico* validation of mNGS-based diagnostics: (i) generation of *in silico* validation data sets that are reflective of real-world clinical specimens with relation to background host and microbial nucleic acid content and (ii) utilization of high-quality genomes with verified taxonomy to ensure accurate characterization of specimens. This is an area where extensively curated and quality-controlled reference databases, such as FDA dAtabase for Reference Grade micrObial Sequences (FDA-ARGOS), can be leveraged.

Bioinformatic reference database composition and curation are critical components of mNGS workflows to ensure accurate results from patient samples. To obtain a sufficient representation of microbial taxa necessary for taxonomic classification of mNGS data, it is necessary to leverage publicly available genomes deposited in repositories, such as the National Center for Biotechnology Information (NCBI). However, sequences deposited in public reference databases are variably curated and may contain misannotations or contamination, which may lead to misclassification, inaccurate results, and potential misdiagnoses (37, 38). Furthermore, no high-quality genomes exist for many organisms, such as rare fungal and parasitic pathogens.

Several public efforts have been made to address misannotation and contamination of public microbial reference sequences, including NCBI Reference Sequence Database (39) and FDA-ARGOS (40), which have sought to impose more stringent quality control criteria for reference sequences included in these databases. Despite these efforts, contamination remains measurable; recent analyses have estimated that approximately 0.34% of sequences in RefSeq exhibit contamination signals, with a disproportionately higher impact on fungal and parasitic genomes (41). Taxonomic underrepresentation is also pronounced, particularly for fungal and parasitic genomes, which are encountered less frequently but are clinically relevant in sterile site infections (42), and the increasing incidences of infections in immunocompromised hosts including patients who have have undergone hematopoietic stem cell or solid organ transplantation (43). Similar limitations are evident in FDA-ARGOS, which, while highly curated, currently contains a limited number of complete genomes relative to the breadth of organisms potentially encountered with mNGS. While compositional issues remain for RefSeq and FDA-ARGOS, these efforts should be commended for highlighting the critical need for stringent quality control and curation of metagenomic reference sequence databases. Others have leveraged approaches to correct false positive classifications by curating poor quality sequence alignments from contaminated sequences in NCBI GenBank as the reference for the database to broadly capture taxonomic diversity (33). In the absence of clear regulatory guidelines governing database inclusion or exclusion criteria, manufacturers and reference laboratories have established their own criteria and approaches to ensure the accuracy of reference databases (2, 3, 33, 38). These approaches highlight the importance of transparency, documentation, and traceability in database construction and maintenance.

Challenges related to reference database quality are not unique to mNGS and have long been recognized in whole-genome sequencing and Sanger sequencing-based microbial identification workflows. However, these limitations may be amplified in mNGS because taxonomic classification depends on large, continuously evolving databases spanning a broad diversity of organisms, including many pathogens that may be encountered infrequently in clinical practice. Moreover, a lack of consensus in the literature or from regulatory bodies to define standardized quality control criteria and the burden of evidence required to demonstrate effective database curation for manufacturers are uniquely challenging for broad mNGS databases. Engagement with regulatory bodies provides a framework for outlining plans to leverage post-market data to validate the performance of these organisms.

### Change management implications

Beyond reference database composition and the process of assembling a high-quality reference database, continual curation and documentation of change control processes remain critical to ensure reproducibility of results and to avoid the accidental introduction of contaminated sequences that could impact diagnostic accuracy. While software lifecycle management, version control, and documentation are well-established requirements for medical device manufacturers (e.g., IEC62304). However, updates to bioinformatics algorithms or reference databases may directly alter the organisms detected, their taxonomic classification, or the reporting thresholds applied during interpretation. Consequently, change management activities must consider not only software functionality but also the potential impact of updates on analytical and clinical performance, which may be difficult to assess without repeating extensive analytical or clinical validation studies.

For mNGS, there is a balance between maintaining reproducibility and supporting the flexibility needed to incorporate new organisms, improved taxonomic classification, and emerging public health threats. To mitigate risks to device and patient safety, a risk-based classification approach aligned with ISO14971 or CLSI EP19-Ed3 (44) may be used by IVD manufacturers and clinical reference laboratories developing mNGS-based LDTs to determine the probability and severity of risk.

Defining standardized algorithms and database update protocols through predetermined change control plans (PCCPs) can serve as an effective tool for risk-based software and database lifecycle management for IVD manufacturers (45). Future frameworks may benefit from a detailed definition of risk-based approaches that distinguish minor updates from modifications likely to alter diagnostic performance. In the absence of mNGS-specific guidance, developers often rely on case-by-case regulatory dialog to define acceptable change management practices.

## FROM SIGNAL TO ACTIONABLE RESULTS: INTERPRETING AND REPORTING CHALLENGES IN MNGS RESULTS

### Interpretation and reporting heterogeneity

Analytic interpretation of clinical metagenomic data to generate actionable results for clinicians is challenging and remains a key translational barrier to the adoption of this technology in clinical practice, particularly outside large reference laboratories with dedicated bioinformatic expertise. While analytical detection of microbial nucleic acids is an essential component of mNGS testing, interpretation remains a major challenge because detection alone does not establish disease causation. As a result, reporting frameworks must balance analytical sensitivity with clinical relevance and provide sufficient transparency regarding confidence, limitations, and uncertainty.

There is substantial heterogeneity in the content and format of mNGS reports, ranging from qualitative, syndromic-focused outputs to quantitative reports containing sequencing and quality-control metrics (35; Tables 1 and 2). Given the diversity of reference databases and bioinformatic pipelines used for analysis, transparency regarding what an assay can and cannot detect is critical for both clinicians and regulators. Communication of quality control metrics, reporting thresholds, and confidence criteria becomes particularly important when extending beyond pathogen identification to applications such as antimicrobial resistance gene detection, where deeper sequencing and higher genome coverage may be required to achieve comparable performance.

**TABLE 2:** Potential clinical reporting elements for mNGS assays^*a*^

Category	Sample element
Organism detection results (i.e., Analytic interpretation)	Taxonomic level reported (species/genus)
	Quantitative or semiquantitative signal (e.g., reads, RPM, rank)
	Confidence or evidence tier/category
	Link to intended use and limitations
Interpretation & clinical context	Specimen-specific interpretive caveats
	Co-infection notes (if applicable)
Antimicrobial resistance (if reported)	Genotypic determinants that are detected
	Definition of interpretation (e.g., reporting genotype vs predicting phenotype)
	Predicted phenotype (if validated against conventional susceptibility testing)
	Coverage/confidence thresholds
Limitations & disclaimers	Definition of reporting scope (inclusivity)
	Organisms not well covered by the database
	Sensitivity limits for low-abundance pathogens
	Known contamination or interpretive risks

^*a*^ Elements shown are examples and should not be interpreted as a recommended or standardized reporting template for all mNGS assays.

Interpretation criteria are also highly specimen-specific. Reporting thresholds appropriate for sterile specimens may differ substantially from those used for non-sterile sample types such as respiratory tract specimens, where distinguishing colonization from infection remains a major challenge (46). Even in sterile samples, contamination introduced during specimen collection or processing may complicate interpretation. Consequently, validation of reporting criteria across diverse real-world samples remains essential for the clinical accuracy and reliability of mNGS results. Future reporting frameworks may benefit from distinguishing between likely pathogens, organisms of uncertain clinical significance, organisms consistent with commensal flora, and findings suspected to represent contamination. Such an approach may provide clinicians with additional context while preserving the broader detection capabilities of mNGS.

### Clinical adjudication and orthogonal confirmation

Because mNGS assays are intended to aid in the diagnosis of infection, results require clinical adjudication. Interpretation may incorporate microbiology findings, imaging studies, histopathology results, host immune status, antimicrobial exposure, and overall clinical presentation. Such multidisciplinary assessment may be particularly important when mNGS identifies unexpected organisms, multiple potential pathogens, or findings that are discordant with conventional diagnostic testing.

Orthogonal confirmation may also be appropriate in selected situations. For example, targeted PCR, culture, serology, or histopathology may be used to support low-abundance detections, unexpected pathogens, organisms commonly associated with contamination, or findings with substantial therapeutic implications. Future regulatory frameworks may benefit from recognizing that not all mNGS findings carry the same level of evidentiary certainty and that predefined approaches for adjudication, confirmation, and reporting of borderline findings may improve consistency across laboratories.

### Appropriate utilization

Appropriate utilization of mNGS represents an important consideration for both clinical implementation and regulatory oversight. The intended use of the assay is shaped by both analytical factors and clinical context and guides the selection of patients and clinical scenarios where broad-range pathogen detection is likely to influence patient management. The clinical value of mNGS tests depends on the test being ordered in the appropriate clinical context, the interpretation of results impacting clinical decision-making (47–50), and a demonstrable effect on patient outcomes.

Current use cases where mNGS may provide particular value include central nervous system infections, culture-negative infections, severe unexplained infections, respiratory failure in critically ill patients, and infections in immunocompromised hosts. In these settings, broad-range pathogen detection may complement conventional diagnostic testing by enabling simultaneous evaluation of bacterial, viral, fungal, and parasitic pathogens from a single assay. At present, mNGS may be best positioned as a complementary or secondary diagnostic tool in selected clinical scenarios, whereas broader first-line implementation will require additional evidence demonstrating clinical utility, cost-effectiveness, and impact on patient outcomes.

Finally, broader adoption of mNGS will likely require alignment among regulators, laboratories, clinicians, and payers regarding appropriate use, interpretation, and clinical utility. Reimbursement for infectious disease mNGS remains sparse and inconsistent, in part, because tests are often applied across heterogeneous patient populations and clinical scenarios, making streamlined coding and demonstration of clinical utility to support coverage challenging. Key regulatory and validation challenges and potential paths forward are summarized in Table 3.

**TABLE 3:** Summary of key regulatory and validation challenges

Challenge	Why existing frameworks struggle	Potential path forward
Representative organism validation	Exhaustive organism-level validation is impractical	Representative organism framework and definition of the burden of evidence required by regulators for representative organism approaches
Contamination and kitome	Agnostic detection amplifies interpretation challenges	Standardized contamination controls and reporting thresholds
Clinical validation	Rare pathogens often lack comparator methods	Alternative comparators and composite reference standards
Database quality	Reference databases are incomplete and continuously evolving	Database curation and transparency standards
Pipeline updates	Software and database changes may alter diagnostic output, but software and databases must have mechanisms for change to adapt to evolving clinical standards and emerging pathogens	Risk-based change control and PCCPs
Intended use	Utility varies across patient populations and the scope of the reporting claims need to be defined so clinicians can use mNGS tests effectively	Defined clinical use cases and intended-use claims
Reimbursement	Clinical utility evidence remains limited	Evidence generation that demonstrates clinical utility across labeled indications

Transparent reporting criteria, including confidence thresholds, assay limitations, and intended use, will be important for consistent interpretation of mNGS results. Future frameworks should also define risk-based approaches for clinical adjudication and orthogonal confirmation of unexpected or clinically significant findings. Clear use cases and evidence demonstrating clinical utility will be essential to support broader regulatory adoption, reimbursement, and clinical implementation.

## TOWARD FIT-FOR-PURPOSE OVERSIGHT OF AGNOSTIC MNGS DIAGNOSTICS

Unlike PCR-based molecular diagnostics, where sample-to-answer integration is often feasible, mNGS workflows are inherently modular, spanning sample preparation, sequencing instrumentation, bioinformatics pipelines, and reference databases. Existing quality frameworks, including CLSI guidance, CAP checklist expectations, and CLIA laboratory oversight, provide useful foundations for documentation, validation, competency, and reporting but do not fully address the agnostic and continuously evolving nature of mNGS diagnostics (51, 52).

**Fig 1:**
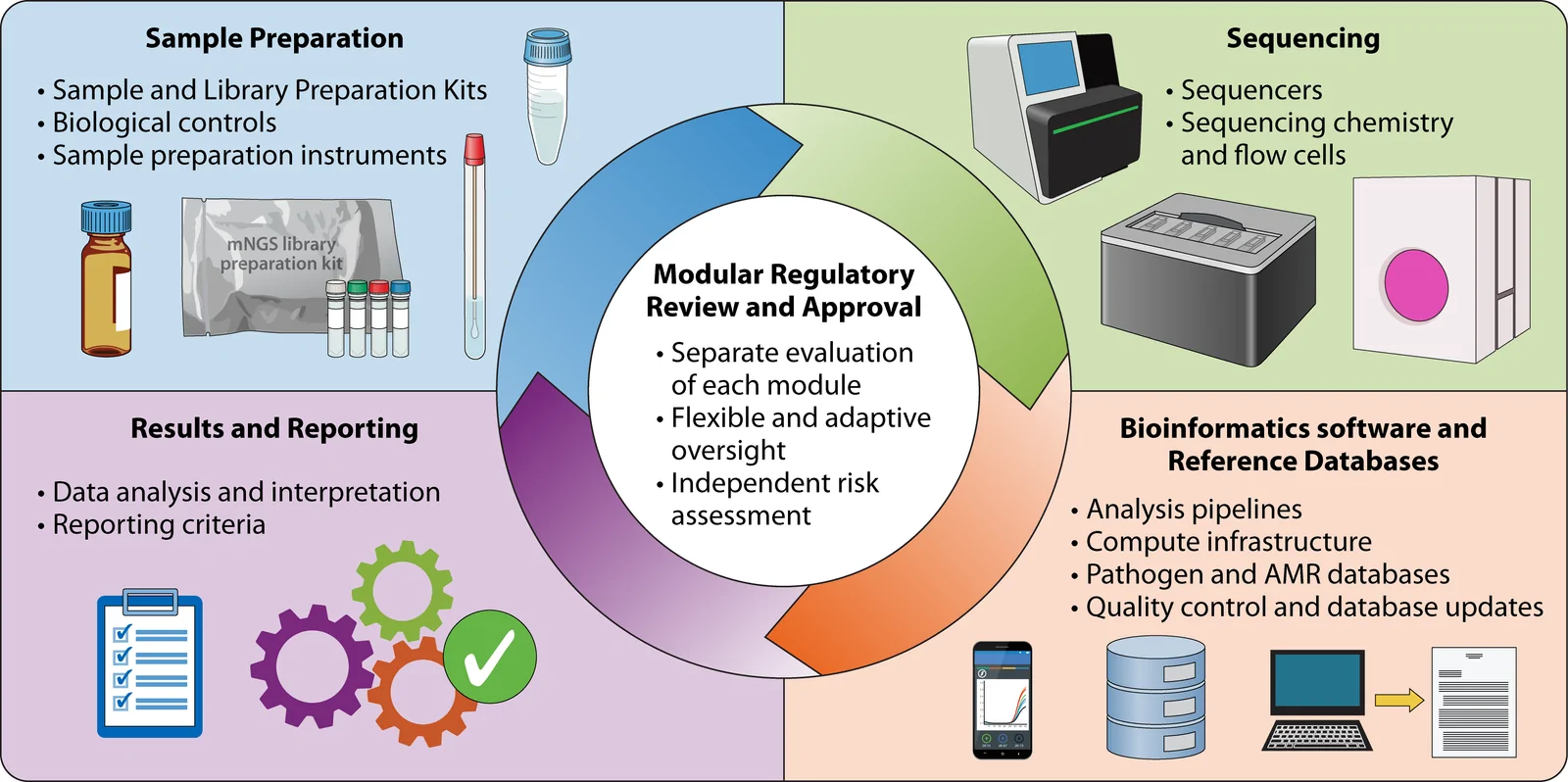
Conceptual modular regulatory model (reagents, instrumentation, software, and databases reviewed as components).

One potential future direction is for regulatory bodies to consider pathways enabling independent review of discrete components, such as reagent kits (library prep, extraction, QC tools), diagnostic-grade sequencing instruments, bioinformatics software regulated as a medical device (SaMD), and reference sequence databases (Fig. 1). Such modular evaluation could reduce the developer burden, support incremental innovation, improve supply chain resilience, and enhance preparedness for public health emergencies. Importantly, examples of modular oversight exist within current regulatory frameworks, including SaMD guidance, instrument-only *de novo* reviews, and PCCPs in antimicrobial susceptibility test systems (53). Extending such concepts to mNGS could improve analytical rigor, avoid redundant validation, and enable specialized regulatory review.

Another useful precedent comes from oncology: diagnostics initially focused on detecting mutations in known oncogenes and tumor suppressor genes, but comprehensive genomic profiling with NGS-based assays has now become the standard, enabling precision medicine approaches to cancer treatment. FDA downgraded the risk level of these diagnostics from Class III to Class II and implemented special controls, such as a detailed description of all somatic mutations intended to be detected by the test. Therefore, FDA authorization of oncology NGS assays is tied to specific clinical claims, such as using a biomarker to select a therapy, rather than to the entire scope of genomic information a test can generate. Broader genomic findings may still be reported for tumor profiling purposes but are often distinguished from findings that support specific labeled clinical claims (54). This intended use framework could be adopted for mNGS-based diagnostics that provide a similar context for infectious diseases.

Importantly, adoption of NGS-based diagnostics in oncology was driven not only by advances in sequencing technology but also by clearly defined clinical use cases and the ability to support therapeutic decision-making. A similar approach may be valuable for infectious disease mNGS. Consensus around priority clinical applications, such as central nervous system infections, culture-negative infections, severe unexplained infections, and infections in immunocompromised hosts, could facilitate the development of intended-use-specific performance expectations, clinical utility studies, reimbursement pathways, and regulatory review. The emergence of FDA-authorized NGS-based infectious disease diagnostics further demonstrates that regulatory pathways for sequencing-based approaches are feasible when the intended use, performance expectations, and clinical claims are clearly defined.

While this discussion focuses on US regulatory pathways, similar challenges exist internationally, including within the European Union’s *In Vitro* Diagnostic Regulation (EU IVDR) framework, where engagement mechanisms for novel diagnostics remain limited. Harmonizing a global regulatory approach through dialog among regulatory bodies will also reduce the burden on manufacturers seeking to market their technology globally.

## CONCLUSION

Agnostic mNGS approaches are reshaping infectious disease diagnostics, yet regulatory and interpretive frameworks have not kept pace with technology’s agnostic potential. This minireview highlights structural mismatches between existing validation paradigms and the realities of mNGS, spanning analytical thresholds, clinical interpretation, and bioinformatic change management. We outline considerations that could support more predictable, scalable oversight without constraining innovation or clinical judgment. Positioned as a resource for dialog among laboratories, developers, regulators, and public health partners, these perspectives aim to facilitate patient access to mNGS-based diagnostics and strengthen preparedness for both routine diagnostics and emerging infectious threats. Future consensus efforts could help translate these considerations into implementation-ready frameworks for mNGS interpretation, regulatory evaluation, reimbursement, and clinical adoption.

## AFTER EPUB

*[This article was published on 10 September 2026 with missing information in the text, Acknowledgments, Conflict of Interest statement, and References. The information was added in the current version, published on 20 October 2026.]*
